# Decrease in Use of Manual Vacuum Aspiration in Postabortion Care in Malawi: A Cross-Sectional Study from Three Public Hospitals, 2008–2012

**DOI:** 10.1371/journal.pone.0100728

**Published:** 2014-06-25

**Authors:** Maria L. Odland, Hanne Rasmussen, Geir W. Jacobsen, Ursula K. Kafulafula, Piaroza Chamanga, Jon Ø. Odland

**Affiliations:** 1 Department of Cancer Research and Molecular Medicine, Norwegian University of Science and Technology, Trondheim, Norway; 2 Department of Public Health and General Practice, Norwegian University of Science and Technology, Trondheim, Norway; 3 Kamuzu College of Nursing, Blantyre, Malawi; 4 Department of Community Medicine, University of Tromsø, Tromsø, Norway; 5 School of Health Systems and Public Health, University of Pretoria, Pretoria, South Africa; Karolinska Institutet, Sweden

## Abstract

**Objectives:**

To investigate the use of manual vacuum aspiration in postabortion care in Malawi between 2008–2012.

**Methods:**

A retrospective cross-sectional study was done at the referral hospital Queen Elisabeth Central Hospital, and the two district hospitals of Chiradzulu and Mangochi. The data were collected simultaneously at the three sites from Feb-March 2013. All records available for women admitted to the gynaecological ward from 2008-2012 were reviewed. Women who had undergone surgical uterine evacuation after incomplete abortion were included and the use of manual vacuum aspiration versus sharp curettage was analysed.

**Results:**

Altogether, 5121 women were included. One third (34.2%) of first trimester abortions were treated with manual vacuum aspiration, while all others were treated with sharp curettage. There were significant differences between the hospitals and between years. Overall there was an increase in the use of manual vacuum aspiration from 2008 (19.7%) to 2009 (31.0%), with a rapid decline after 2010 (28.5%) ending at only 4.9% in 2012. Conversely there was an increase in use of sharp curettage in all hospitals from 2010 to 2012.

**Conclusion:**

Use of manual vacuum aspiration as part of the postabortion care in Malawi is rather low, and decreased from 2010 to 2012, while the use of sharp curettage became more frequent. This is in contrast with current international guidelines.

## Introduction

Malawi is a small landlocked country in South-Eastern Africa, and is one of the poorest countries in the world [1]. The health system is deprived, with limited resources and number of medical doctors [1]. Malawi has among other serious issues one of the highest maternal mortality rates (MMR) in the world [2]. The MMR has decreased over the past years, but the numbers are still high, and every year 3,000 women die from pregnancy-related causes [2].

An unsafe abortion is defined as a “Procedure for terminating an unintended pregnancy either by individuals without the necessary skills or in an environment that does not conform to minimum medical standards, or both” [3]. Globally, of about 20 million unsafe abortions are performed every year, 47,000 women die as a consequence [4]. The majority of unsafe abortions take place in developing countries, with the heaviest burden in Africa [5]. Studies indicate that 24–30% of all maternal mortality in Malawi is caused by unsafe abortions [6], [7]. This is because induced abortion is highly restricted and is only legal in order to save a pregnant womańs life [7], [8]. Thus, it is inaccessible for the majority of women [7], [9].

In Malawi there are many unwanted pregnancies due to a number of reasons such as lack of contraception and family planning, contraception failure, poverty, and shame [10]. In spite of the strict abortion law, in 2009 it was estimated that 18,700 women were treated for complications from induced abortion in health facilities in Malawi [11]. Including women who did not receive treatment and those who did not have any abortion complications, the estimated number of induced abortions was about 70,000 every year, and most of these were unsafe [11]. Complications that may occur are incomplete abortion, sepsis, haemorrhage, uterine perforation, chronic pain, infertility and death [9], [12]. It may be hard to distinguish complications from induced versus spontaneous abortions, as the symptoms can be similar [12], [13]. Additionally, shame and fear of being punished, among others, may make women conceal that the abortion was induced.

An assessment done by the World Health Organization (WHO), International Projects Assistance Services (IPAS), and the Ministry of Health in Malawi has summarized four key recommendations that may reduce the MMR in Malawi: reviewing the restrictive abortion laws, strengthening the national family planning programme, addressing sexual and reproductive health needs of young Malawians, and strengthening postabortion care (PAC) services [7]. PAC is a concept of interventions introduced by IPAS in the early 1990s. It includes treatment for complications from spontaneous or induced abortion, provision of postabortion contraception, family planning and other health services [14].

Complications from abortion are treated in different ways depending on severity and type. Incomplete abortion with retained products of conception (RPOC) in the uterus may occur, with anaemia or infections as a consequence. RPOC is generally treated with uterine evacuation (UE), either surgically or medically [15]. Surgical evacuation involves vacuum aspiration, either manual (MVA) or electrical (EVA), or dilatation and sharp curettage (D&C) [15]. MVA is done using a hand-held syringe, while EVA is done with an electric or foot-operated mechanical pump [15]. D&C on the other hand is mostly performed in an operating theatre, often under general anaesthesia, and a sharp metal curette is used to evacuate the uterine contents [15].

According to the WHO, vacuum aspiration should be the primary method in surgical evacuation of the uterus after incomplete abortions in the first trimester of pregnancy, or up to fourteen weeks by a skilled provider [16]. It is faster, less painful, can be done with less haemorrhage than D&C [16] and performed with local anaesthesia or analgesia. Because sedation and electricity are not necessary, MVA can be done on an outpatient-basis and in places where resources and equipment are less available. It is thus suitable for settings like public hospitals in Malawi. Midwives and nurses can also carry out the procedure in areas where the number of medical doctors is limited. As a consequence, in addition to giving fewer complications than D&C, MVA saves both time and resources [15], [16].

Medical management with Misoprostol is a good option that is also recommended by the WHO and is becoming more common worldwide. Recent studies from other low resource settings comparable to Malawi have shown that Misoprostol is a good option to MVA [17], [18] in first trimester abortions, but may not be applicable in all settings due to limited resources and availability. Additionally, medical abortion is rarely used, partly because of reluctant clinicians [19].

An assessment done in Malawi in 1999 found that most hospitals offered only D&C to treat incomplete abortions [20]. As a result of the study, a Malawi PAC intervention program was implemented in many hospitals in the early 2000s. It focused on training and upgrading of facilities, with increased use of MVA as one of the aims [20].

A report from 2009 [21] showed that more than half of UEs (55%) were done with MVA, 44% with D&C, while less than 2% of patients got medical or some other treatment [21]. Private hospitals more often used MVA (77%) than public hospitals (50%) [21]. The lower use of MVA compared to D&C [21] is in contrast to the current international recommendations [16] and despite that it is held as an incorporated commodity on the government's standard list of equipment [22]. There are many possible reasons why MVA is not used. According to the 2009 report, there was either no available equipment or it was worn out. Further, care providers lacked training. In some places the equipment was locked in to prevent it from being used to induce abortions [7], [21].

We wanted to study the use of MVA in public health facilities in Malawi. As the most recent data on the subject were collected in 2009 [7], [21], our study aimed to investigate if there had been a change in use over the years that followed. Updated knowledge may have an impact on the development and improvement of PAC routines, and thereby help the authorities on their way to improve maternal health and reduce the MMR.

## Methods

### Study design and setting

The study was designed to ascertain the use of MVA in women who were afflicted by complications of incomplete abortion at three different hospitals in Malawi. We chose a retrospective and cross-sectional study design.

The study was conducted at the gynaecological wards of three public hospitals in Malawi. They were Mangochi District Hospital, Chiradzulu District Hospital, and Queen Elisabeth Central Hospital (QECH) in Blantyre.

The majority of PAC cases are treated in public hospitals [21]. The two district hospitals were selected because there were indicators of poor maternal health within their catchment areas. Such indicators include little use of contraceptives, which may contribute to higher rates of abortion. The current use of modern contraceptives is around 27% and 46% for Mangochi and Chiradzulu districts, respectively [23]. QECH was chosen because it is a referral hospital for the southern region of Malawi and as such, admits cases including complications from incomplete abortions in the districts of the southern region.

### Study population

The study targeted all women who were diagnosed with incomplete abortion and admitted to the three hospitals for surgical evacuation with D&C or MVA between 2008 and 2012.

### Inclusion/Exclusion criteria

We reviewed all patient records that could be identified from January 2008 to December 2012 at the three study facilities. We included all women who were treated with UE due to complications after incomplete abortion. Records of women who were admitted for other reasons were not included. Since complications of spontaneous and induced abortions often are difficult to distinguish [13], these were not separated. Women who were admitted, but did not need or receive surgical evacuation were not included in the study. So were women where UE had been planned, but where it was insufficiently documented that the procedure had been carried out. Currently, foetal death up to 28 weeks of gestation is considered an abortion in Malawi due to lack of resources at the neonatology department. Therefore, all incomplete abortions before gestational age (GA) of 28 weeks were included in the study.

### Study period

Data collection was conducted in February and March 2013.

### Study sample

Based on vital statistics of the female population of childbearing age, variations in health behaviour, characteristics of the study hospitals and other health facilities in the region, and difficulties to distinguish between spontaneous and induced abortions, we estimated that we would be able to identify about 8,000 cases.

All available files covering each year at the three gynaecological departments were reviewed and 5,162 files from women who received UE after incomplete abortion were identified. Women receiving medical treatment were only recorded at QECH (Oxytocin (n = 4) or Misoprostol (n = 14)). The low numbers compared favourably with the number of medical abortions found in 2009 [21]. Due to the limited number and the insufficient information in the files, we did not include these women in our study. Women who had received other treatment like antibiotics and bed rest were also not included. EVA was not available at any of our three hospitals. When medical treatment (mostly oxytocin) was given in addition to surgical evacuation (n = 62), we considered surgery as the primary treatment and included these cases in their respective group as either D&C or MVA. Some cases with no information about year of hospital stay (n = 23) were also excluded. This left us with 5,121 cases.

### Data collection

Three different teams of health personnel, either midwives or 5th year medical students, who were familiar with medical terms, reviewed patient records at each facility. The teams collected data concurrently at each site. Prior to data collection all teams received instructions from the same local supervisor, and all used the same data extraction tool. We collected information about type of evacuation, duration of hospital stay, and whether the woman had been referred from another health facility. Furthermore, demographic data (residence, age, tribe, marital status, level of education, religion, and occupation) and obstetric data (gravity, parity, number of live children, and gestation at the time of abortion) were also recorded.

### Statistical analysis

Data were analysed using IBM SPSS Statistics version 20 (Armonk, New York, USA). Values were given as proportions (percent) with 95% confidence interval (CI) of 95%. The number of procedures was compared by hospital and year (2008–2012) using chi-square statistics.

### Ethics statement

Before data collection, the local College of Medicine Research and Ethics Committee (COMREC) in Blantyre granted approval. Permission to assess the patient records was also sought and granted by the District Medical Officers at Mangochi and Chiradzulu district hospitals and the director of QECH. All patient information was anonymized and de-identified prior to analysis.

## Results

The demographic characteristics of the women included by year are given in Table 1. Overall mean age was 24.9 (SD 6.5) years. Many women had given several births, with the maximum parity of 12, while 29.1% (95% CI, 27.6–30.6) had no previous births. The majority were married (85.8%; 95% CI, 84.4–87.3). The proportion of women with higher education was 2.8% (95% CI, 1.7–4.3), and 66.0% (95% CI, 38.7–68.1) did not have paid work.

**Table 1:** Demographic characteristics of women who received treatment for retained products of conception after incomplete abortion in three hospitals in Malawi, 2008–2012^a^.

Characteristics		2008 (n = 1075)	2009 (n = 1091)	2010 (n = 593)	2011 (n = 722)	2012 (n = 1640)
Median age; year (range)		24 (9–50)	24 (11–52)	24 (13–44)	24 (12–47)	24 (11–50)
Pregnancy history	Primigravida	183 (23.4)	188 (24.3)	121 (28.3)	87 (22.5)	301 (25.6)
	Multigravida	598 (76.6)	585 (75.7)	306 (71.7)	300 (77.5)	875 (74.4)
Number of living children	None	233 (34.5)	215 (36.1)	137 (39.1)	98 (36.8)	366 (54.7)
	1	164 (24.3)	114 (19.1)	73 (20.9)	46 (17.3)	129 (19.3)
	≥ 2	278 (41.2)	267 (44.8)	140 (40.0)	122 (45.9)	174 (26.0)
Gestational age	1^st^ trimester	399 (38.4)	378 (36.3)	225 (38.9)	224 (33.4)	548 (35.7)
	2^nd^ trimester	571 (55.0)	601 (57.7)	305 (52.7)	402 (59.9)	878 (57.2)
	3^rd^ trimester	68 (6.6)	63 (6.0)	49 (8.5)	45 (6.7)	109 (7.1)
Religion	Roman Catholic	38 (11.3)	39 (13.4)	30 (14.8)	55 (22.0)	116 (18.2)
	Anglican	41 (12.2)	16 (5.5)	20 (9.9)	9 (3.6)	14 (2.2)
	Church of Central Africa	85 (25.4)	47 (16.2)	35 (17.2)	31 (12.4)	87 (13.7)
	Pentecostal	61 (18.2)	15 (5.2)	11 (5.4)	19 (7.6)	15 (2.4)
	Seventh Day Adventist	18 (5.4)	16 (5.5)	17 (8.4)	10 (4.0)	39 (6.1)
	Islam	65 (19.4)	106 (36.6)	51 (25.1)	64 (25.6)	112 (17.6)
	Church of God	8 (2.4)	16 (5.5)	14 (6.9)	16 (6.4)	74 (11.6)
	Other	19 (5.7)	35 (12.1)	25 (12.3)	46 (18.4)	179 (28.1)
Marital Status	Unmarried^b^	78 (13.5)	57 (16.0)	39 (13.7)	32 (12.9)	119 (14.4)
	Married	498 (86.5)	299 (84.0)	245 (86.3)	217 (87.1)	709 (85.6)
Hospital admittance^c^	Rural facility	537 (50.0)	836 (75.6)	333 (56.2)	536 (74.2)	709 (43.2)
	Urban facility	538 (50.0)	255 (23.4)	260 (43.8)	186 (25.8)	931 (56.8)
Educational level	None	125 (39.4)	42 (31.1)	63 (43.8)	18 (36.0)	11 (15.3)
	Primary	66 (20.8)	37 (27.4)	40 (27.8)	18 (36.0)	30 (41.7)
	Secondary	117 (36.9)	54 (40.0)	40 (27.8)	13 (26.0)	24 (33.3)
	Tertiary	9 (2.8)	2 (1.5)	1 (0.7)	1 (2.0)	7 (9.7)
Occupation	None	137 (26.9)	43 (18.3)	69 (30.4)	51 (23.1)	553 (61.6)
	Housewife	147 (28.8)	94 (40.0)	91 (40.1)	83 (37.6)	112 (12.5)
	Student	66 (12.9)	35 (14.9)	23 (10.1)	17 (7.7)	47 (5.2)
	Gainful employment^d^	160 (31.4)	63 (26.8)	44 (19.4)	70 (31.7)	186 (20.7)

(n = 5121)

a Numbers are given as n (%) unless otherwise indicated

b Unmarried: Single, separated, divorced, widowed.

c Rural: Chiradzulu and Mangochi Hospital. Urban: QECH

d Gainful employment; Cleaner, Farmer, Businesswoman, Policewoman and other.

The use of MVA between 2008–2012 is given in Tables 2 and 3. The overall use in the three study hospitals (n = 5,121) was 17.3% (95% CI, 16.2–18.3) (Table 2). Correspondingly, surgical evacuation with sharp curettage was used in 82.7% (95% CI, 81.3–83.4) of the cases. Table 3 shows 1^st^ trimester abortions only. Of all the incomplete abortions in the 1^st^ trimester 34.2% were treated with MVA (Table 3).

**Table 2:** Surgical methods used for removal of retained products of conception after incomplete abortion by year and hospital in three hospitals in Malawi, 2008–2012^a^.

Year	Method used	Chiradzulu (n = 1632)	Mangochi (n = 1319)	QECH^c^ (n = 2170)	All hospitals (n = 5121)
2008 (n = 1075)	MVA^d^	44 (18.3)	11 (3.7)	157 (29.2)	212 (19.7)
	D&C^e^	196 (81.7)	286 (96.3)	381 (70.8)	863 (80.3)
2009 (n = 1091)	MVA^d^	38 (11.2)	231 (46.5)	69 (27.1)	338 (31.0)
	D&C^e^	301 (88.8)	266 (53.5)	186 (72.9)	753 (69.0)
2010 (n = 593)	MVA^d^	17 (8.0)	64 (52.9)	88 (33.8)	169 (28.5)
	D&C^e^	195 (92.0)	57 (47.1)	172 (66.2)	424 (71.5)
2011 (n = 722)	MVA^d^	10 (3.0)	57 (28.2)	19 (10.2)	86 (11.9)
	D&C^e^	324 (97.0)	145 (71.8)	167 (89.8)	636 (88.1)
2012 (n = 1640)	MVA^d^	11 (2.2)	36 (17.8)	34 (3.7)	81 (4.9)
	D&C^e^	496 (97.8)	166 (82.2)	897 (96.3)	1559 (95.1)
All years (n = 5121)	MVA^d^	120 (7.4)	399 (30.3)	367 (16.9)	886 (17.3)
	D&C^e^	1512 (92.6)	920 (69.7)	1803 (83.1)	4235 (82.7)

All trimesters^b^.

a Numbers are given as n (%) unless otherwise indicated.

b Gestational age up to 28 weeks.

c Queen Elisabeth Central Hospital (QECH).

d Manual Vacuum Aspiration (MVA).

e Dilatation and sharp Curettage (D&C).

**Table 3:** Surgical methods used for removal of retained products of conception after incomplete abortion by year and hospital in three hospitals in Malawi, 2008–2012^a^.

Year	Method used	Chiradzulu (n = 582)	Mangochi (n = 454)	QECH^c^ (n = 738)	All hospitals (n = 1774)
2008 (n = 399)	MVA^d^	28 (32.9)	9 (9.0)	135 (63.1)	172 (43.1)
	D&C^e^	57 (67.1)	91 (91.0)	79 (36.9)	227 (56.9)
2009 (n = 378)	MVA^d^	21 (18.6)	128 (74.4)	47 (50.5)	196 (51.9)
	D&C^e^	92 (81.4)	44 (25.6)	46 (49.5)	182 (48.1)
2010 (n = 225)	MVA^d^	12 (15.2)	39 (92.9)	75 (72.1)	126 (56.0)
	D&C e	67 (84.8)	3 (7.1)	29 (27.9)	99 (44.0)
2011(n = 224)	MVA^d^	7 (6.0)	37 (54.4)	12 (30.0)	56 (25.0)
	D&C^e^	109 (94.0)	31 (45.6)	28 (70.0)	168 (75.0)
2012 (n = 548)	MVA^d^	9 (4.8)	25 (34.7)	22 (7.7)	56 (10.2)
	D&C^e^	180 (95.2)	47 (65.3)	265 (92.3)	492 (89.8)
All years (n = 1774)	MVA^d^	77 (13.2)	238 (52.4)	291 (39.4)	606 (34.2)
	D&C^e^	505 (86.8)	216 (47.6)	447 (60.6)	1168 (65.8)

Abortions in 1^st^ trimester^ b^.

a Numbers are given as n (%) unless otherwise indicated.

b Gestational age up to 12 weeks.

c Queen Elisabeth Central Hospital (QECH).

d Manual Vacuum Aspiration (MVA).

e Dilatation and sharp Curettage (D&C).

**Figure 1:**
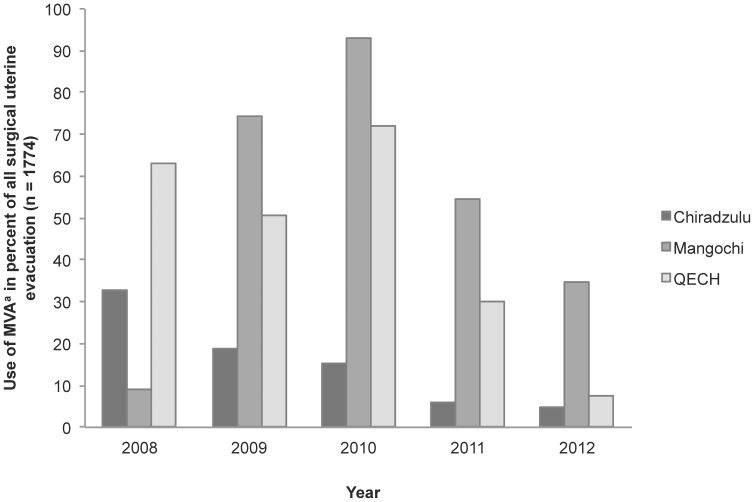
Use of manual vacuum aspiration for removal of retained products of conception after incomplete abortions in three hospitals^a^ in Malawi between 2008–2012. First trimester^b^. ^a^ Queen Elisabeth Central Hospital (QECH), Mangochi District Hospital, Chiradzulu District Hospital. ^b^ Up to 12 weeks of gestation.

There was an apparent increase in the use of MVA from 2008 (19.7%; (95%CI, 17.4–22.2) to 2009 (31.0%; 95% CI 28.3–33.8), with a rapid decline after 2010 (28.5%; 95% CI, 24.9–32.3) down to only 4.9% (95% CI, 3.9–6.1) in 2012. Fig. 1 shows the rapid decline that occurred simultaneously at all the three study hospitals during 2010–2012. Comparing the first of the study period (2008–2010) and the second (2011–2012), the MVA use was significantly lower at the end of the study period (p<0.001).

There were significant differences between the hospitals. Overall, the highest use of MVA was found at the district hospital of Mangochi (30.3%; 95% CI, 27.78–32.81) with Chiradzulu at the lower end (7.4%; 95% CI 6.13–8.73). However, differences between hospitals varied by year (Table 2 and 3).

Where information was available (n = 4,865) the mean gestational age (GA) among women was slightly higher at QECH (mean 13.3 weeks SD 5.1) than at the two other hospitals (Chiradzulu; mean 12.4 weeks SD 5.5, Mangochi; mean 12.5 weeks SD 5.5). The use of MVA was higher in first trimester abortions (34.2%), compared to second (9.3%) and third (0.6%) trimester abortions.

## Discussion

We studied the use of MVA compared to sharp curettage in cases of incomplete abortions in three public hospitals in Malawi, and found that the frequency varied over the years and between the studysites. Only 34.2% of 1^st^ trimester incomplete abortions were treated with MVA between 2008–2012. The use of MVA decreased after 2010, and the rate was clearly lower in 2012 than in the four preceding years, while the use of sharp curettage increased.

A major source of bias may be that by use of a retrospective design, the study did not cover all the records of women admitted due to complications from abortion. This was mostly due to lack of quality of the archive system of the selected hospitals. Especially at QECH many files had been lost. According to hospital employees, some patient records had been thrown away due to lack of space. Additionally, patients might have taken their records home or to the pharmacy. Other records may have been taken away and used for teaching purposes. These are all reasons why we found less than the 8,000 files we anticipated, and may explain the range in numbers of records by year, i.e. range 593 (2010) to 1,640 (2012).

Records were stored in boxes for each month, with no system by patient names or diagnosis. Many records were of poor quality with incomplete data. Most of them had information about age, GA and the PAC method that was used. Unfortunately, rather few files held information on complications or patient outcome. Importantly, no maternal deaths were identified. These records were said to be stored elsewhere, destroyed, or misplaced because they had been used for teaching purposes. Bearing in mind the mortality statistics from unsafe abortions, a substantial number of women most likely died during the study period [9], [24].

By basing the study on patient records the care providers did not know the documents would be subjected to research. This may have prevented information bias and the probability of influencing the management provided.

Three different teams collected data at the respective study hospitals and this could be a source of misclassification. However, the teams were trained by the same supervisor prior to data collection. Additionally, the same data extraction tool was used in order to prevent such information bias.

Since the study took place in just three hospitals in southern Malawi, it may not be representative of the whole country. Nevertheless, the study provides new information that the respective hospitals may use to improve their PAC services.

Compared to our results for the year 2009 (Table 3) a previous concurrent study found a higher use of MVA in 1^st^ trimester abortions (51.9% versus 72.4%) [21]. However, the latter study did not have a retrospective design, it included both private and public facilities and the study period was only 30 days. The low use of medical abortion or with Cytotec (1.4%) was however quite similar to our findings [21].

There were quite big differences in use of MVA between the hospitals. There may be several reasons for this. QECH is a referral hospital and therefore could have received patients who were transferred from health centres or other hospitals after unsuccessful attempts of MVA, or patients with severe complications. Other explanations could be differences in resources, staff turnover and number of skilled clinicians or perhaps the hospitals used different manners to implement MVA in the routines.

As mentioned earlier a source of bias is the amount of missing files. However, in QECH, the central hospital, a higher proportion of records from 2012 were available (n = 931) due to improvements in the archive system. Hence, we consider the 2012 data from QECH of fairly good quality. MVA was used in 7.7% compared to D&C in 92.3% of 1^st^ trimester abortions. To us it seems clear that this cannot be explained by missing records, and we consider these results quite alarming, especially because it is the central hospital in the region. It may also indicate that the same trend seen in the other hospitals is not pure coincidence.

We found few women who received single medical treatment. Studies have confirmed that Misoprostol is a good option and that it should be given greater attention in case the Malawian authorities attempt to improve PAC [19]. We did not aim to investigate this any further. However, some doctors at QECH revealed that they do not use medical abortion because it takes time to see the result of the treatment. The procedure may also fail to evacuate the uterus. In a crowded gynaecological ward one may rather use a method that is well known and works immediately. Hence, doctors tend to go straight to evacuation of the uterus with sharp curettage.

The study was not designed to find the reason for any decreasing use of MVA. We asked a few nurses and doctors about it. Some claimed that using MVA gave more complications, and that persons who had been trained were not working there anymore. Others said that the equipment was not intact and that lack of resources was the main barrier. This is a probable explanation, as Malawi has experienced an economic crisis since 2009 [25]. Problems with PAC equipment running out of stock was mentioned in a report as early as 2004 [20], indicating that there were logistic issues rather than shortage of equipment in central medical stores [20]. Similar issues have been discussed in other developing countries [19], [26]. A previous report mentioned that equipment was locked away in fear of misuse [7]. Similar explanations may also be the case for medical management of incomplete abortion. Future studies exploring this might be helpful to change the routines in a longer perspective.

This study is not necessarily valid for all developing countries, however it points out several important aspects that contributes to high MMR in low resource settings. PAC intervention programs have been implemented in many developing countries, but we should ask ourselves if it has led to changes that are sustainable in the health care systems where they were applied.

Maternal mortality from unsafe abortions is a significant international problem that can be dealt with [9]. Experience and evidence from other countries, for example South Africa, Bangladesh and Romania have showed that maternal mortality can be reduced by a change of legislation towards abortion on demand [27]. However, in Malawi abortion is not legal, and if legalized, the health care system would still need resources and time to be able to offer safe abortion services. At the moment other measures must therefore be found to reduce morbidity and mortality from abortion complications.

By choosing MVA or medical treatment instead of sharp curettage whenever possible, maternal morbidity and mortality can be reduced. The hospitals can also save resources that the public health system would find useful in other areas.

## Conclusion

We studied the use of MVA versus sharp curettage to treat RPOC in incomplete abortions in Malawi. Use of MVA was lower than recommended by the WHO and decreased considerably from 2009 to 2012. Conversely, sharp curettage became more frequent. This is in contrast with international guidelines. The Ministry of Health in Malawi and public health authorities should act upon the findings with appropriate interventions. The increased use of sharp curettage instead of MVA should be investigated, including prospective monitoring of clinical practice. Possible ways forward could be educational sessions for clinicians and making equipment more available. Another suggestion is that authorities try to implement new routines for retaining patient records.

If the results from this study are acted upon, a proportional increase in use of MVA may reduce maternal morbidity and mortality in Malawi.
